# Evaluating the Diagnostic Utility of Cystatin C versus Creatinine in Chronic Kidney Disease: A Cross-Sectional Analysis

**DOI:** 10.7759/cureus.98573

**Published:** 2025-12-06

**Authors:** Pallavi Sagar, Ravi Shekhar, Bandana Kumari, Prit P Singh, Praveen Kumar

**Affiliations:** 1 Biochemistry, All India Institute of Medical Sciences, Patna, IND; 2 Biochemistry, Indira Gandhi Institute of Medical Sciences, Patna, IND; 3 Nephrology, Indira Gandhi Institute of Medical Sciences, Patna, IND; 4 General Medicine, Indira Gandhi Institute of Medical Sciences, Patna, IND

**Keywords:** biomarkers, chronic kidney disease, creatinine, cystatin c, early diagnosis, egfr

## Abstract

Background: Chronic kidney disease (CKD) is a progressive condition with high prevalence in India, often driven by diabetes and hypertension. Early detection is critical, yet serum creatinine, the conventional biomarker, is influenced by non-renal factors and lacks sensitivity in early stages. Cystatin C, a low molecular weight protein unaffected by muscle mass or diet, has emerged as a promising alternative. This study evaluates the diagnostic utility of serum cystatin C versus creatinine in detecting and monitoring CKD among Indian patients.

Methods: A cross-sectional observational study was conducted at Indira Gandhi Institute of Medical Sciences, Patna, over 18 months, enrolling 150 participants: 75 patients with CKD (grades 3-5) and 75 healthy controls. Serum cystatin C was measured using an enzyme-linked immunosorbent assay and serum creatinine via the kinetic Jaffe method. Estimated glomerular filtration rate (eGFR)_Cr-Cys_, eGFR_Cr_, and eGFR_Cys_ were calculated using Chronic Kidney Disease Epidemiology Collaboration or CKD-EPI equations based on both biomarkers (serum creatinine and serum cystatin C). Statistical analyses were conducted using t-tests, chi-square tests, and Pearson correlation coefficients to compare biomarker levels and assess their relationship with eGFR.

Results: Both serum cystatin C and creatinine were significantly elevated in patients with CKD compared to controls (p<0.0001). Cystatin C showed a stronger negative correlation with eGFR_Cr-Cys_ (r = -0.91) than creatinine (r = -0.896). A high concordance (r = 0.90) was observed between eGFR values derived from cystatin C and creatinine equations in patients with CKD, particularly in diabetic subgroups and early-stage CKD. In healthy controls, this correlation was weaker and non-significant.

Conclusion: Serum cystatin C is a sensitive and reliable biomarker for CKD detection, demonstrating superior correlation with eGFR_Cr-Cys_ compared to creatinine. Its diagnostic performance is especially valuable in early-stage disease and high-risk populations. Integrating cystatin C into routine clinical practice could enhance early diagnosis and improve patient outcomes, though cost and assay availability remain barriers.

## Introduction

Chronic kidney disease (CKD) is a progressive disorder defined by structural or functional kidney abnormalities persisting for more than three months [1, 2]. It is a major global health burden, with rising prevalence linked to diabetes, hypertension, and cardiovascular disease [3, 4]. The kidneys regulate waste excretion, fluid balance, and hormone secretion, functions best reflected by the glomerular filtration rate (GFR).

While exogenous markers such as inulin clearance provide accurate GFR, they are costly and impractical. Serum creatinine, the most common endogenous marker, is inexpensive but influenced by muscle mass, diet, age, and drugs, making it insensitive for early CKD [5]. This has led to interest in cystatin C, a low molecular weight protein produced by all nucleated cells, freely filtered at the glomerulus, and unaffected by muscle or diet. Evidence suggests it detects renal dysfunction earlier than creatinine [6, 7].

In India, CKD prevalence is estimated at 800 per million, with ESRD incidence of 150-200 per million. Diabetes and hypertension account for nearly two-thirds of cases, but additional risk factors such as low birth weight, nutritional deficiencies, chronic infections, and obstructive uropathies contribute significantly. Nearly 28% of Indian children are born underweight, predisposing them to reduced nephron number and lower baseline GFR. Access to renal replacement therapy is limited; over 90% of patients needing dialysis die prematurely, and many discontinue treatment due to cost. This makes early detection particularly critical in the Indian context [8].

Diabetes induces glomerular hyperfiltration and sclerosis, while hypertension accelerates vascular and glomerular injury. Together, they synergistically hasten CKD progression. Genetic predispositions (e.g., *UMOD* and *APOL1*) also influence susceptibility. Importantly, the kidney compensates for nephron loss, delaying symptoms until nearly half of renal function is lost. This silent progression underscores the inadequacy of creatinine as a late marker and highlights the potential of cystatin C for earlier detection [9].

Cystatin C is produced at a constant rate, freely filtered, and almost completely metabolized by proximal tubules. Its levels are unaffected by muscle mass, gender, or diet. Studies show a stronger correlation with measured GFR than creatinine, especially in elderly, diabetic, or low-muscle-mass populations [10]. Equations such as the Chronic Kidney Disease Epidemiology Collaboration (CKD-EPI) cystatin C improve GFR estimation, alone or combined with creatinine [11]. Despite this, creatinine remains dominant in practice due to cost and limited assay availability.

Although global evidence supports cystatin C, Indian data are limited. Most studies rely on creatinine-based equations, which may underestimate CKD in low-muscle-mass populations common in India. The combined impact of diabetes and hypertension on cystatin C in Indian patients is also underexplored. Given the high CKD burden and socioeconomic barriers to treatment, validating cystatin C as a sensitive biomarker could enable earlier diagnosis and intervention, improving outcomes.

Superiority of eGFR estimation using both cystatin and creatinine is well known. However, in some resource-limited settings, it is not possible to access the gold standard diagnostic. Clinicians and biochemists still have doubts about how close cystatin C or creatinine can be to the true eGFR. This doubt necessitated the need for this study. 

This study seeks to address the research question of whether cystatin C is a more sensitive and reliable biomarker than creatinine for detecting and monitoring CKD in Indian patients. The central hypothesis is that serum cystatin C levels are significantly elevated in CKD compared to healthy controls and demonstrate a stronger correlation with estimated GFR (eGFR) than creatinine, thereby offering superior potential for early detection of renal dysfunction. To test this hypothesis, the study aims to measure serum cystatin C and creatinine levels in both patients with CKD and controls, compare eGFR values derived from cystatin C- and creatinine-based equations, evaluate the correlations of cystatin C with creatinine and eGFR, and ultimately determine the utility of cystatin C as an alternative biomarker for early CKD detection in the Indian population.

## Materials and methods

Study overview

This was a cross-sectional, observational study conducted over a period of 18 months, from February 2021 to October 2022. The study was carried out in the Department of Biochemistry in collaboration with the Departments of Nephrology and General Medicine at Indira Gandhi Institute of Medical Sciences (IGIMS), Patna. Ethical approval was obtained from the Institutional Ethics Committee (vide letter no. 1936/IEC/IGIMS/2020), and the study adhered to the principles of the Helsinki Declaration and Good Clinical Practice guidelines. Written informed consent was obtained from all participants before enrolment.

Study population

The study population consisted of two groups. The cases were patients diagnosed with CKD attending the Department of Nephrology at IGIMS. Controls were age- and sex-matched healthy individuals visiting the Department of Medicine for routine check-ups, with no known renal disease.

Inclusion criteria included adults aged >18 years with a confirmed diagnosis of CKD (for cases) and healthy volunteers (for controls). Exclusion criteria included patients with acute kidney injury, rapidly progressive glomerulonephritis, acute-on-CKD, active infection, renal malignancies, congenital anomalies of the kidney and urinary tract, and solitary kidney, patients on dialysis, and those with recent exposure to nephrotoxic drugs.

Sample size

A total of 150 participants were enrolled, comprising 75 patients with CKD (grades 3-5) and 75 healthy controls. The sample size was determined based on the expected difference in biomarker levels between cases and controls, ensuring 85% statistical power to detect significant associations at 0.05 alpha value.

Data collection

Demographic and clinical details, including age, sex, and comorbidities such as diabetes mellitus and hypertension, were recorded using a structured proforma. Venous blood samples (5 mL) were collected from all participants under aseptic precautions. Samples were allowed to clot at room temperature for 20-30 minutes and centrifuged at 3000 rpm for 10 minutes. The separated serum was analyzed immediately or stored at -20°C until further testing. Hemolyzed samples were excluded.

Methodology

**Figure 1:**
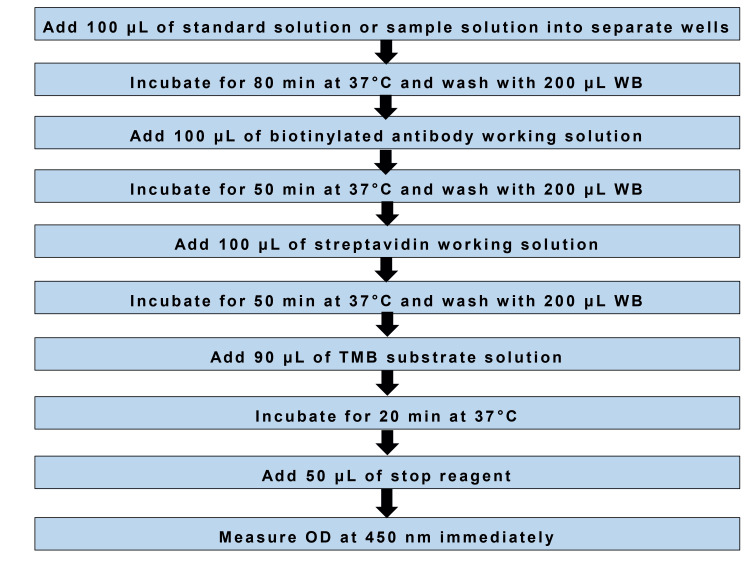
Assay procedure for serum cystatin C OD: Optical density; TMB: Tetramethylbenzidine; WB: Wash buffer.

Two biochemical parameters were measured in this study: serum cystatin C and serum creatinine. Serum cystatin C was estimated using a sandwich enzyme-linked immunosorbent assay (ELISA). Micro-ELISA plates pre-coated with anti-human cystatin C antibody were employed for the assay. Serum samples were incubated with a biotinylated detection antibody followed by streptavidin-horseradish peroxidase conjugate. After thorough washing to remove unbound components, tetramethylbenzidine substrate was added to initiate the colorimetric reaction. The reaction was terminated with an acidic stop solution, and absorbance was measured at 450 nm. The concentration of cystatin C in each sample was then determined by comparing the optical density values against a standard calibration curve (Figure 1).

Serum creatinine was measured using the kinetic Jaffe method. In this procedure, creatinine reacts with alkaline picrate to form a yellow-orange complex. The rate of change in absorbance at 520/800 nm was directly proportional to the concentration of creatinine in the sample. To ensure accuracy and reproducibility, daily calibration was performed, and appropriate quality control materials were analyzed alongside patient samples using standard reagents.

The eGFR was calculated for all participants using both the CKD-EPI creatinine equation and the CKD-EPI cystatin C equation. eGFR_Cr-Cys_, eGFR_Cr_, and eGFR_Cys_ were calculated using CKD-EPI equations based on both biomarkers (serum creatinine and serum cystatin C). These calculated values were compared to assess the diagnostic performance of cystatin C relative to creatinine in evaluating renal function. This approach allowed for a direct comparison of the sensitivity and reliability of the two biomarkers in detecting CKD.

Outcome parameters

The primary outcome parameters included mean serum cystatin C levels and mean serum creatinine in patients with CKD versus controls and correlation of cystatin C and creatinine with eGFR_Cr-Cys_. Secondary outcomes included comparison of eGFR values derived from cystatin C and creatinine and subgroup analysis of biomarker levels in patients with diabetes, hypertension, or both.

Statistical analysis

Data were entered into Microsoft Excel (Microsoft Corp., Redmond, WA, USA) and analyzed using Graph Pad version 8.4.3 (Dotmatics, Boston, Massachusetts). Continuous variables were expressed as mean ± standard deviation. Differences between cases and controls were assessed using the unpaired Student’s t-test. Categorical variables were compared using the chi-square test. Correlation between serum cystatin C, serum creatinine, and eGFR was evaluated using Pearson’s correlation coefficient. A p-value of <0.05 was considered statistically significant.

## Results

A total of 150 participants were enrolled in the study, comprising 75 patients with grade 3-5 CKD and 75 healthy controls. The mean age was slightly higher in the patient group (51.87 years) compared to the control group (49.95 years), and a greater proportion of participants were male in both groups. However, the p-values for both age (0.18) and gender (0.51) indicate that these differences were not statistically significant (Table 1).

**Table 1:** Age and gender distribution of cases and controls *Unpaired t-test (t = 1.350); **Fisher’s exact test. SD: Standard deviation.

Parameter	Cases (n = 75)	Controls (n = 75)	p-value
Age in years, mean ± SD	51.87 ± 7.35	49.95 ± 9.88	0.18*
Male gender, n (%)	46 (61.3%)	42 (56.0%)	0.51**

The majority of patients (48.00%) had both hypertension and diabetes, and within this group, a very high proportion (91.67%) had severe kidney impairment (eGFR <30 mL/min/1.73 m^2^). While a greater percentage of patients with both conditions fell into the severe CKD category compared to those with only diabetes or hypertension, the p-value of 0.13 indicates that this distribution pattern was not statistically significant, meaning the presence of these comorbidities alone did not show a significant association with the severity of eGFR reduction in this sample (Table 2).

**Table 2:** Distribution of patients with respect to eGFR in diabetes mellitus and hypertension CKD: Chronic kidney disease; eGFR: Estimated glomerular filtration rate.

Grade of CKD	Total cases (%)	eGFR <30 mL/min/1.73 m^2^ (n = 62)	eGFR ≥30 mL/min/1.73 m^2^ (n =13)	Chi-square value	p-value (chi-square)
Diabetes mellitus (%)	22 (29.33)	16 (72.73)	6 (27.27)	4.007	0.13
Hypertension (%)	17 (22.67)	13 (76.48)	4 (23.52)		
Hypertension and diabetes mellitus (%)	36 (48.00)	33 (91.67)	3 (8.33)		

The results show that both biomarkers were significantly elevated in the CKD group compared to controls, with a mean difference of 2.23 mg/L for cystatin C and 2.53 mg/dL for creatinine. The 95% confidence intervals for these differences do not include zero, and the extremely low p-values (<0.0001) confirm that these elevations are statistically highly significant, clearly demonstrating that both serum cystatin C and creatinine are strongly associated with the presence of CKD (Table 3).

**Table 3:** Comparison of serum cystatin C and serum creatinine between patients with CKD and controls CKD: Chronic kidney disease; CI: Confidence interval.

Parameter	CKD cases (n = 75)	Controls (n = 75)	Difference in mean	95% CI of difference	t-value	p-value (unpaired t-test)
Serum cystatin C (mg/L)	2.96 ± 0.90	0.73 ± 0.05	2.23	2.03–2.43	26.39	<0.0001
Serum creatinine (mg/dL)	3.36 ± 1.02	0.83 ± 0.15	2.53	2.30–2.77	21.25	<0.0001

**Figure 2:**
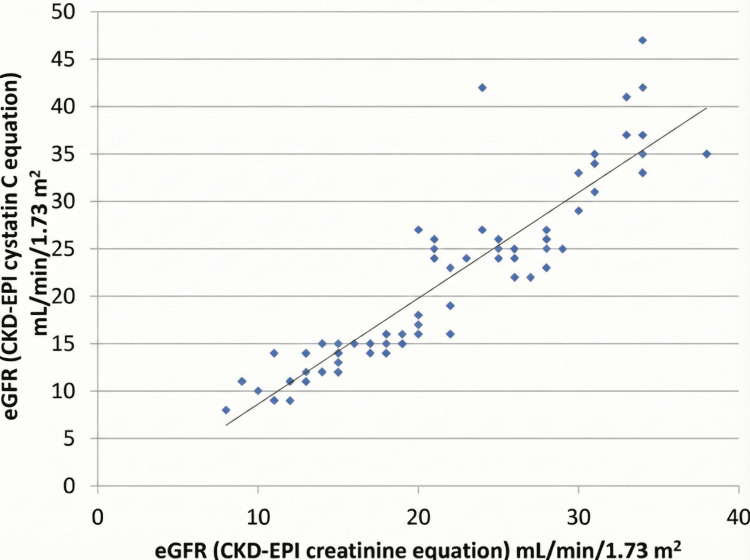
Scatter diagram showing Pearson correlation analysis between eGFR (CKD-EPI cystatin C equation) and eGFR (CKD-EPI creatinine equation) in patients with CKD Linearity of the correlation is represented in the equation: eGFR (CKD-EPI cystatin equation) = 1.116*eGFR (CKD-EPI creatinine equation) - 2.532. CKD-EPI: Chronic Kidney Disease Epidemiology Collaboration; eGFR: Estimated glomerular filtration rate.

In patients with CKD, there was a very strong positive correlation between serum cystatin C and serum creatinine (r = 0.90), and both biomarkers showed a strong negative correlation with eGFR_Cr-Cys_ (r ≈ -0.90), indicating that as eGFR decreases, both biomarker levels increase. Furthermore, the two eGFR equations (based on cystatin C and creatinine) were highly correlated in patients with CKD (r = 0.90) and in diabetic/non-diabetic subgroups, but this correlation was weaker and non-significant in healthy controls (r = 0.62, p>0.05). This suggests that while these markers and equations are excellent and concordant for assessing renal function in disease states, their relationship is less consistent in healthy individuals (Table 4, Figure 2).

**Table 4:** Correlation analyses of biomarkers and eGFR in patients with CKD and controls CI: Confidence interval; CKD-EPI: Chronic Kidney Disease Epidemiology Collaboration; eGFR: Estimated glomerular filtration rate.

Correlation pair (variables)	Study group	Correlation coefficient (r)	R²	95% CI	p-value
Serum cystatin C vs. serum creatinine	CKD patients	0.90	0.82	0.86 to 0.94	<0.001
Serum cystatin C vs. eGFR_Cr-Cys_	CKD patients	–0.91	0.83	–0.95 to –0.87	<0.001
Serum creatinine vs. eGFR_Cr-Cys_	CKD patients	–0.896	0.80	–0.93 to –0.84	<0.001
eGFR (CKD-EPI cystatin C eqn.) vs. eGFR (CKD-EPI creatinine eqn.)	CKD patients	0.90	0.82	0.85 to 0.94	<0.001
Serum cystatin C vs. serum creatinine	Controls	0.62	0.39	0.47 to 0.75	<0.05
eGFR (CKD-EPI cystatin C eqn.) vs. eGFR (CKD-EPI creatinine eqn.)	Controls	0.62	0.39	0.47 to 0.75	>0.05
eGFR (CKD-EPI cystatin C eqn.) vs. eGFR (CKD-EPI creatinine eqn.)	Diabetic CKD patients	0.92	0.85	0.80 to 0.97	<0.001
eGFR (CKD-EPI cystatin C eqn.) vs. eGFR (CKD-EPI creatinine eqn.)	Non-diabetic CKD patients	0.90	0.81	0.84 to 0.94	<0.001
eGFR (CKD-EPI cystatin C eqn.) vs. eGFR (CKD-EPI creatinine eqn.)	CKD patients with eGFR <30 mL/min/1.73 m^2^	0.84	0.70	0.74 to 0.90	<0.001
eGFR (CKD-EPI cystatin C eqn.) vs. eGFR (CKD-EPI creatinine eqn.)	CKD patients with eGFR ≥30 mL/min/1.73 m^2^	0.91	0.83	0.67 to 0.97	<0.001

## Discussion

This study was conducted with the primary objective of evaluating the utility of serum cystatin C as a biomarker for the detection of CKD, in comparison to the conventional marker, serum creatinine. The results clearly demonstrate that serum cystatin C is a highly sensitive and reliable marker for renal dysfunction, particularly in the early stages of CKD.

The study found that both serum cystatin C and creatinine levels were significantly elevated in patients with CKD compared to healthy controls, with a strong positive correlation (r = 0.90) between the two biomarkers in the patient group. This indicates that cystatin C performs comparably to creatinine in identifying renal impairment. However, cystatin C showed a stronger negative correlation with eGFR_Cr-Cys_ (r = -0.91) than creatinine (r = -0.896), suggesting it may be more sensitive to changes in GFR. This is consistent with previous studies, such as those by Dharnidharka et al. and Mussap et al., which also reported cystatin C to be a superior marker for early GFR decline [12, 13].

A key finding was the strong concordance between eGFR values calculated using cystatin C and creatinine-based equations (r = 0.90) in patients with CKD, supporting the use of cystatin C as an alternative or complementary tool for GFR estimation. This aligns with studies by Mohd Tahir et al. and Madero et al., who also found high agreement between the two methods [14, 15]. Notably, in healthy controls, this correlation was weaker and non-significant, underscoring the clinical utility of cystatin C primarily in diseased populations.

The study also stratified patients based on diabetes and hypertension, key risk factors for CKD. Although diabetic CKD patients had a lower mean eGFR than non-diabetics, the difference was not statistically significant. However, cystatin C-based eGFR showed a stronger correlation with creatinine-based eGFR in diabetic subgroups (R² = 0.85) than in non-diabetics (R² = 0.81), reinforcing its reliability in high-risk groups. This is supported by Pucci et al. and Zhang et al., who highlighted cystatin C’s diagnostic efficiency in diabetic nephropathy [16, 17].

Furthermore, in patients with early-stage CKD (eGFR ≥30 mL/min/1.73 m2), cystatin C exhibited a stronger correlation with eGFR_Cr-Cys_ (R² = 0.83) than in those with advanced disease (R² = 0.70), suggesting its particular value in detecting mild renal impairment. This finding is consistent with research by Rinno et al., which also emphasized cystatin C’s sensitivity in early CKD [18].

In summary, this study confirms that serum cystatin C is a robust and early biomarker for CKD, especially in populations where creatinine measurements may be influenced by non-renal factors such as muscle mass, age, or diet. Its strong correlation with established measures of renal function and its performance in high-risk and early-stage patients with CKD support its integration into routine clinical practice for improved detection and monitoring of kidney disease.

A primary limitation of this study is the absence of a direct comparison with a gold-standard method for measuring GFR, such as inulin, iohexol, or chromium-51 ethylenediaminetetraacetic acid clearance. While this is a common constraint in clinical studies due to the cost, invasiveness, and complexity of gold-standard methods, it limits the ability to definitively state cystatin C's superiority over creatinine in reflecting the true GFR. Future research should aim to incorporate these direct measurement techniques, even in a smaller subset of participants, to validate the diagnostic accuracy of serum cystatin C against an unimpeachable reference. This would provide a more robust foundation for its adoption and help refine the cystatin C-based eGFR equations for the specific demographic profile of the study population.

From a clinical practice perspective, a significant barrier identified is the current lack of standardization and widespread availability of cystatin C testing, coupled with its higher cost compared to the deeply entrenched and inexpensive creatinine assay. A key future direction involves health economic studies to demonstrate that the long-term benefits of earlier CKD detection and intervention with cystatin C, such as reduced progression to end-stage renal disease and lower healthcare costs, outweigh the initial higher test cost. Concurrently, efforts should be made to develop more affordable and automated cystatin C assays and to integrate them seamlessly into existing clinical laboratory workflows, making the test more accessible for widespread screening, particularly in high-risk groups such as diabetics and hypertensives.

## Conclusions

Based on the findings of this study, it can be conclusively stated that serum cystatin C serves as a highly sensitive and reliable biomarker for the detection and evaluation of CKD, demonstrating a strong correlation with serum creatinine and a superior inverse relationship with eGFR in patients WITH CKD. The high concordance between cystatin C-based and eGFR_Cr-Cys_ validates cystatin C as a robust alternative for assessing renal function, particularly in the critical early stages of the disease, where traditional creatinine measurement lacks sensitivity. Therefore, the integration of serum cystatin C into clinical practice, especially as an adjunctive test in high-risk populations such as diabetics and hypertensives, can significantly enhance the early diagnosis and monitoring of CKD, ultimately enabling timely interventions to slow disease progression and improve patient outcomes.
